# Tissue is the issue-sarcoidosis following ABVD chemotherapy for Hodgkin's lymphoma: a case report

**DOI:** 10.1186/1752-1947-1-148

**Published:** 2007-11-25

**Authors:** Vivek Subbiah, Uyen K Ly, Amer Khiyami, Timothy O'Brien

**Affiliations:** 1 Division of Hematology/Oncology, Department of Medicine, Case Western Reserve University School of Medicine, MetroHealth Medical Center, Cleveland, Ohio, USA; 2 Department of Pathology, Case Western Reserve University School of Medicine, MetroHealth Medical Center, Cleveland, Ohio, USA; 3 Department Of Internal Medicine/Pediatrics, Case Western Reserve University School of Medicine, MetroHealth Medical Center, Cleveland, Ohio, USA

## Abstract

Thirty two year old Caucasian female presented 2 months post partum with fevers, cough and shortness of breath. CT scan of the chest to rule out pulmonary embolism revealed mediastinal lymphadenopathy. Biopsy of the nodes revealed classic Hodgkin's lymphoma and she received ABVD chemotherapy. She was in remission as confirmed by a PET/CT scan. Five months later she had another PET/CT scan which showed areas of hypermetabolism indicating a possible relapse. Biopsy revealed sarcoidosis. She received steroids and 18 months later remained in clinical remission. This rare case of sarcoid following classic Hodgkin's lymphoma illustrates that clinical presentation, physical exam, lab investigations and even PET/CT scans may not be able to discriminate between Hodgkin's lymphoma and sarcoidosis. Tissue biopsy and pathological diagnosis remain the gold standard.

## Case presentation

**Figure 1:**
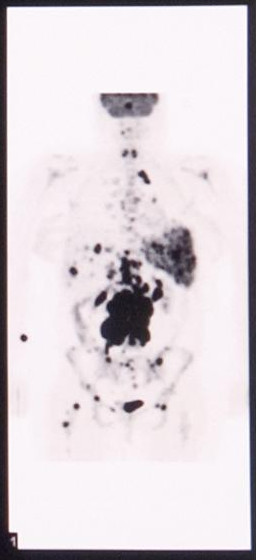
PET/CT scan showing extensive areas of abnormal hypermetabolism in the mediastinum, subcarinal, left hilum, porta hepatis, celiac, retrocrural and superior mesenteric artery nodal areas. In addition, there is increased uptake in the spleen and liver.

**Figure 2:**
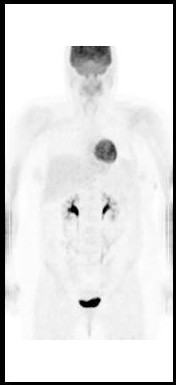
PET/CT showing complete remission.

**Figure 3:**
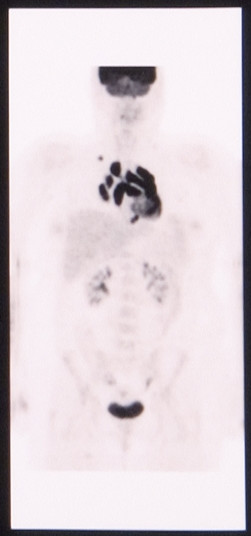
PET/CT showing extensive hypermetabolic mediastinal adenopathy.

**Figure 4:**
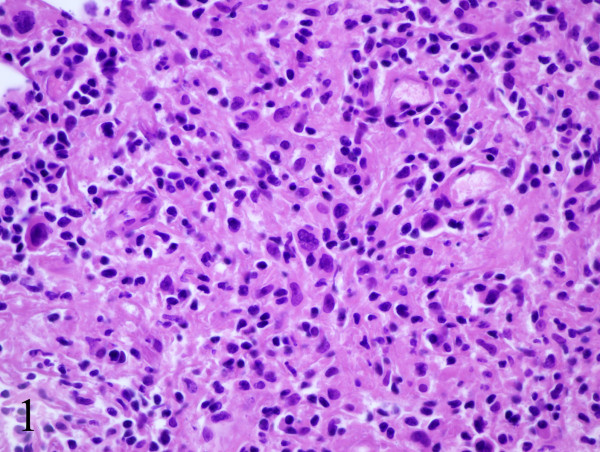
Reed-Sternberg cell variants surrounded by small lymphocytes hematoxylin-eosin stain, original magnification ×40.

**Figure 5:**
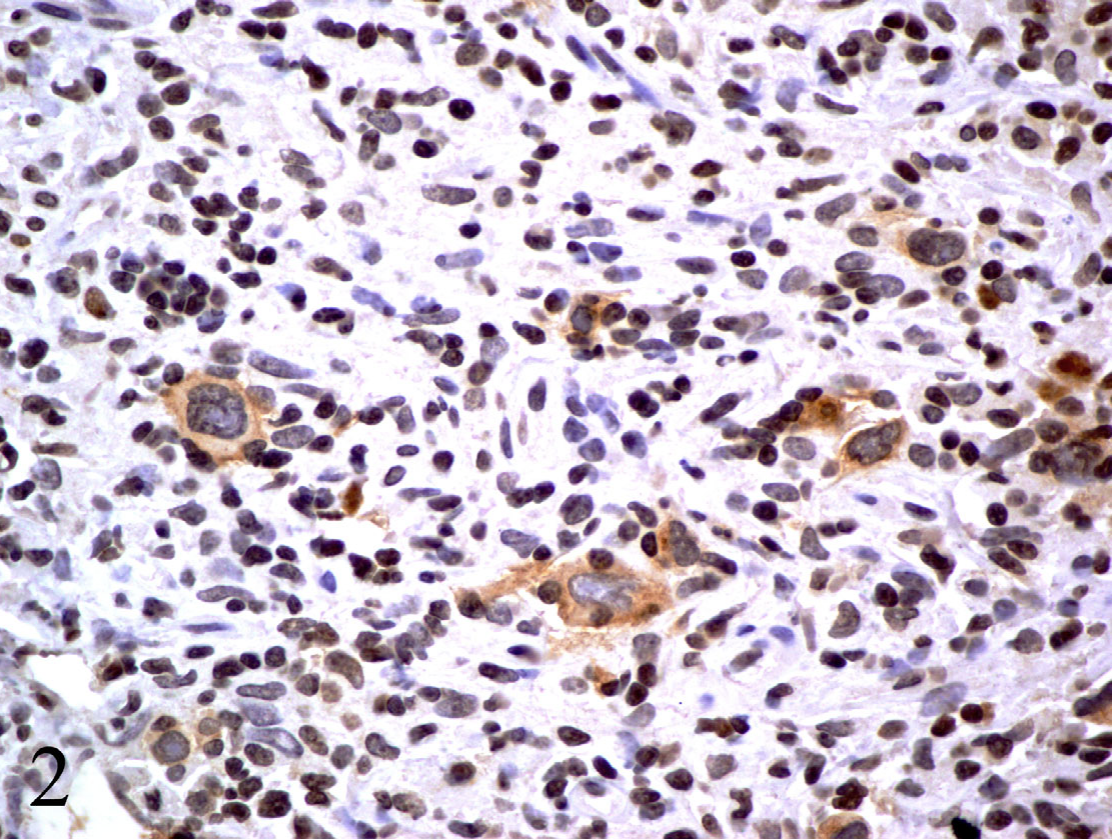
CD30 positive Reed-Sternberg cell variants, original magnification ×40, consistent with classical Hodgkin's lymphoma.

**Figure 6:**
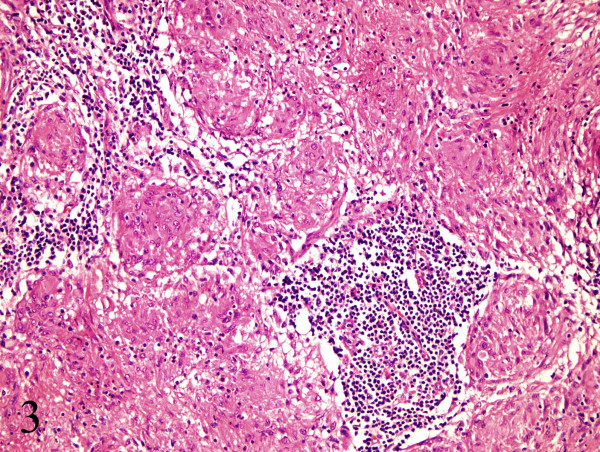
Multiple non-necrotizing, epithelioid granulomas, hematoxylin-eosin stain, original magnification ×40.

A thirty two year old Caucasian female presented two months post partum with high fevers, a dry cough and shortness of breath. CT scan of the chest revealed mediastinal lymphadenopathy and splenomegaly. Subsequent CT scan of the abdomen and pelvis revealed multiple hypodensities in the liver, along with marked periaortic and pericaval lymphadenopathy. CT/PET scan showed extensive areas of abnormal hypermetabolism in the mediastinum, subcarinal, left hilum, porta hepatis, celiac, retrocrural and superior mesenteric artery nodal areas. In addition, there was increased uptake in the spleen and liver. (Figure 1). An initial CT guided core biopsy of a retroperitoneal node was non-diagnostic. A laparoscopic excisional node biopsy and liver biopsy were then performed. Both of these specimens showed involvement with Hodgkin's lymphoma (Figure 4: Reed-Sternberg cell variants surrounded by small lymphocytes hematoxylin-eosin stain, original magnification ×40; Figure 5: CD30 positive Reed-Sternberg cell variants, original magnification ×40, all consistent with classical Hodgkin's lymphoma). She was then treated with standard ABVD (doxorubicin, bleomycin, vinblastine, and dacarbazine). After four cycles she was felt to be in complete remission by PET/CT (Figure 2), then underwent two more cycles and was followed closely. She did well for the next 5 months but then developed neck pain and fatigue. Her physical examination was negative and laboratory evaluation, including a sedimentation rate and LDH, was unremarkable. Her symptoms resolved spontaneously. Three weeks later, she presented with complaints of lower extremity edema and tender erythematous nodules in her lower extremity and wrists which were felt to be consistent with a diagnosis of erythema nodosum. She denied having any pulmonary symptoms, fevers, chills or sweats. A chest x-ray incidentally obtained as part of annual employee health screening for a prior tuberculosis exposure showed new right hilar adenopathy. A PET/CT done to evaluate this revealed extensive hypermetabolic mediastinal adenopathy (Figure 3). With recurrence of Hodgkin's lymphoma in mind, salvage chemotherapy was scheduled and options for stem cell transplant were also discussed. In order to be certain of the diagnosis, the patient underwent a mediastinoscopy. Excisonal biopsies of 3 mediastinal nodes (2 right paratracheal and one subcarinal node) showed numerous non-necrotizing granulomas composed of epithelioid histiocytes, Langhans giant cells and lymphocytes. Ziehl-Neelsen and Gomori Methanamine Silver stains were negative for mycobacteria and fungi. There was no evidence of Hodgkin's lymphoma. The mediastinal node findings were felt to be consistent with a diagnosis of sarcoidosis (Figure 6: Multiple non-necrotizing, epithelioid granulomas, hematoxylin-eosin stain, original magnification ×40). Her provisionally scheduled chemotherapy was cancelled. She was treated with low dose (20 mg/d) prednisone for her erythema nodosum, felt to probably arise as a component of sarcoidosis. Within a few days the tender nodules on her legs resolved completely. Eighteen months later she remains in clinical remission, with 2 follow-up PET/CT scans which were negative for recurrent Hodgkin's lymphoma.

## Discussion and conclusion

Sarcoidosis is a multisystem disorder of unknown etiology characterized by non-caseating granulomas [1]. The diagnosis is established by clinical presentation and confirmed by typical histology. In the USA it has a predilection towards females and in blacks. A sarcoidosis-lymphoma association has been described in which there is an increased incidence of lymphoma at least 5.5 times higher than expected in patients with sarcoidosis. [2] Hodgkin's lymphoma following a diagnosis of sarcoidosis is well reported in the literature[3] and concomitant lymphoma and sarcoidosis have also been described[4]. However, very few reports exist of sarcoid like reactions following treatment of Hodgkin's lymphoma[5]. It has been postulated that bleomycin or other chemotherapeutic agents may precipitate a granulomatous reaction and the development of sarcoidosis but this has not been proven [6,7]. Sarcoidosis often presents with constitutional symptoms such as fever, fatigue, malaise and weight loss but erythema nodosum may also be seen. Hodgkin's lymphoma may present with similar findings but the diagnosis relies on pathological confirmation. Treatment options for relapsed Hodgkin's lymphoma include salvage chemotherapy regimens and/or high dose chemotherapy followed by a stem cell transplant. Since these therapies are potentially very toxic, a definitive tissue confirmation of relapsed Hodgkin's is essential. Clinical presentation, physical exam, lab investigations and, as this case illustrates, even PET/CT scans may not be able to discriminate between Hodgkin's lymphoma and sarcoidosis. Tissue biopsy and pathological diagnosis remain the gold standard.

## Abbreviations

ABVD : Doxorubicin, Bleomycin, Vinblastine, and Dacarbazine chemotherapy

PET/CT : Positron Emission Tomography/Computed Tomography

## Competing interests

The author(s) declare that they have no competing interests.

## Authors' contributions

All authors have read and approved the final manuscript.

## Consent

Informed consent was obtained from the patient for the publication of this case report.
